# Supplementary material for: HIVprotI: an integrated web based platform for prediction and design of HIV proteins inhibitors
Source: J Cheminform. 2018 Mar 9;10:12. doi: 10.1186/s13321-018-0266-y (PMC5845081; doi:10.1186/s13321-018-0266-y)
Supplement: Supplementary file 2 — Additional file 2. Source code of HIVProtI web server. [file 13321_2018_266_MOESM2_ESM.zip › HIVprotI_Source-code/hivprotI/weka-3-6-6/doc/weka/core/converters/DatabaseConverter.html]

DatabaseConverter


---


|  |  |  |
| --- | --- | --- |
| **Overview** \| **Package** \| **Class** \| **Tree** \| **Deprecated** \| **Index** \| **Help** \| **Weka's home** | |  |
| **PREV CLASS**   **NEXT CLASS** | **FRAMES**    **NO FRAMES**     **All Classes** |  |
| SUMMARY: NESTED \| FIELD \| CONSTR \| METHOD | DETAIL: FIELD \| CONSTR \| METHOD |  |


---


## weka.core.converters Interface DatabaseConverter

**All Known Implementing Classes:**: DatabaseLoader, DatabaseSaver

---

``` public interface DatabaseConverter ```

Marker interface for a loader/saver that uses a database

**Version:**
:   $Revision 1.0 $

**Author:**
:   Mark Hall

---

| **Method Summary** | |
| --- | --- |
| `java.lang.String` | `getUrl()` |
| `java.lang.String` | `getUser()` |
| `void` | `setPassword(java.lang.String password)` |
| `void` | `setUrl(java.lang.String url)` |
| `void` | `setUser(java.lang.String user)` |

**Method Detail**

### getUrl

```
java.lang.String getUrl()
```

---


### getUser

```
java.lang.String getUser()
```

---


### setUrl

```
void setUrl(java.lang.String url)
```

---


### setUser

```
void setUser(java.lang.String user)
```

---


### setPassword

```
void setPassword(java.lang.String password)
```


---


|  |  |  |
| --- | --- | --- |
| **Overview** \| **Package** \| **Class** \| **Tree** \| **Deprecated** \| **Index** \| **Help** \| **Weka's home** | |  |
| **PREV CLASS**   **NEXT CLASS** | **FRAMES**    **NO FRAMES**     **All Classes** |  |
| SUMMARY: NESTED \| FIELD \| CONSTR \| METHOD | DETAIL: FIELD \| CONSTR \| METHOD |  |


---
